# The Role of Varicella Zoster Virus (VZV) in Central Nervous System Infectious Syndromes

**DOI:** 10.1155/cjid/6664417

**Published:** 2024-11-13

**Authors:** Viktoria Yakovenko, Pnina Ciobotaro, Rita Bardenstein, Margalit Zusev, Oren Zimhony

**Affiliations:** ^1^Infection Diseases Department, Kaplan Medical Center, Rehovot, Israel; ^2^Clinical Microbiology Laboratory, Kaplan Medical Center, Rehovot, Israel; ^3^Faculty of Medicine, Hebrew University of Jerusalem, Jerusalem, Israel

## Abstract

**Background:** We describe the proportion of VZV infection in central nervous system (CNS) infectious syndromes in a single Israeli medical center.

**Methods:** An observational cohort study was conducted in Kaplan Medical Center (a secondary hospital, Israel) between July 1, 2014, and March 31, 2019. Included were adult patients (≥ 16 years old) with CNS infection with an aseptic CSF profile that were subjected to molecular tests for herpes viruses, HSV either 1 or 2, VZV, enteroviruses, and IgM for West Nile virus (WNV).

**Results:** Clinical presentation suggestive of CNS infection led to lumbar puncture and CSF analysis in 1500 patients yielding 178 cases with aseptic CSF profile. For 62/109 (55.9%) cases of meningitis, the etiology remained unknown, enterovirus accounted for 21 cases (18.9%), VZV for 16 (14.4%), and HSV 1 for 7 (6.3%). One case each of bacterial, fungal, and parainfluenza virus accounted for the other 3 cases. For 30/63 (47.6%) cases of encephalitis, the etiology remained unknown, HSV accounted for 11 cases (17.5%), VZV for 8 (12.7%), and WNV for 10 (15.9%); in two cases, enterovirus was identified and one case of influenza A and one of mycoplasma, accounted for the rest. In six patients with myelitis VZV was identified in 4 (66.7%). Notably, a typical herpetic rash was identified in only 11/28 (39.28%) of cases of VZV CNS infection.

**Conclusions:** VZV is a significant cause of viral CNS infections. In the majority of patients with neurologic syndrome and evident VZV there is no association with a typical herpetic rash. These results apply to Israeli population and likely to other populations with similar background of VZV past infections.

**Summary**

• VZV is a significant viral cause of CNS infections; most VZV CNS infections are not associated with the typical herpetic rash VZV.

## 1. Introduction

Viruses are the major cause of CNS infectious syndromes, meningitis, encephalitis, and myelitis [1–5]. With the introduction of PCR to common practice as a major diagnostic tool for the detection of viral infections involving the CNS, it was realized that the vast majority of viral CNS infections are caused by a few viruses, VZV is one of them [1, 3, 5–10]. The prevalence of CNS infections caused by VZV varies in different studies: (18.5%) [11], (15%) [1], and (11.4%) [6] for encephalitis and meningitis alike. The absence of typical herpetic rash at the time of acute CNS infection due to reactivation of VZV, namely “zoster sine herpete” was noted frequently [2, 4, 5, 7–9, 12–14]. VZV was the most common cause of viral CNS infectious syndromes, encephalitis meningitis, and myelitis in a large collaborative study from Finland from 1995-1996. However, this study included children as well [15].

We describe the proportion of VZV identification amongst other common viral causes in adult patients who presented primarily with CNS infection syndromes, meningitis, encephalitis, and myelitis, with aseptic CSF profile subjected for molecular tests for common viral causes. The study was performed in an Israeli medical center. The presence of herpetic rash at the time of neurologic VZV-related illness was evaluated.

## 2. Materials and Methods

An observational study was conducted at Kaplan Medical Center, Israel (a secondary 550 bed hospital, serving a community of 500,000 people). Molecular test results for VZV were collected between July 1, 2014, and March 31, 2019. A total of 1519 tests were performed.

• Patient information was obtained from the electronic record for age, gender, chronic diseases, clinical signs, neuroimaging tests, treatment, days of hospitalization, and final outcome
• CSF was obtained for analysis at physician discretion for the following combination of symptoms and signs that define these syndromes and aseptic CSF (negative CSF smear only, before culture result) was subject to molecular diagnosis for common viral agents.
  i. Meningitis: Illness characterized by a combination of clinical findings of headache, neck stiffness, photophobia, fever (usually), and evidence of inflammation of the meninges defined by pleocytosis in the spinal fluid.
  ii. Encephalitis: A change in mentation, altered state of consciousness, and or focal signs due to an inflammatory process involving the brain parenchyma with or without laboratory proof.
  iii. Myelitis: Focal clinical neurologic findings with evidence for spinal cord inflammation.

### 2.1. Laboratory Analysis

A PCR testing was performed for the identification of HSV-1 and -2 or VZV [RealStar alpha Herpesvirus PCR Kit 1.0 (ALTONA), sensitivity 95%], enterovirus [RealTime applied Biosystems REF AM1005. Elucigene REF TF005B4. GeneXpert Enterovirus Xpert (Cepheid). Sensitivity 96.3%]. Serology test of CSF for the identification of WNV IgM [Dx select REF EL0300M, Focus], Agglutinin grouping test [CrAg LFA REF CR2003. Sensitivity-from serum-100%, plasma-98.9%, whole blood-99.3%, CSF-100%] for *Cryptococcus*. CSF and blood bacterial culture.

We excluded cases of younger than age 16, cases with no information in the patients' medical records that provided sufficient information for justifying CSF analysis.

## 3. Results

**Figure 1:**
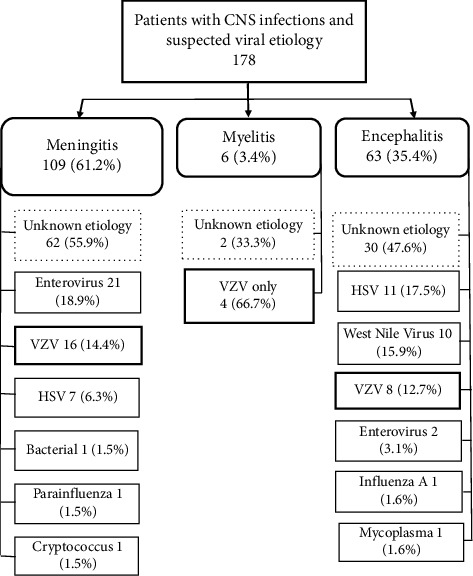
Flowchart of patients with CNS infections and suspicion of viral etiology.

A total of 178 patients with inflammatory neurologic syndromes and CSF profile of aseptic meningitis were analyzed during the 5-year period. The allocation of the CSF analysis to the different syndromes is presented in Figure 1. The characteristics of each case are presented in Table 1, and the main features of the different syndromes are summarized in Table 2.

### 3.1. Syndrome Classification and Herpetic Skin Rash

#### 3.1.1. Meningitis

109 patients (61%) were diagnosed with meningitis, 63 patients (35.4%) had a diagnosis of encephalitis, and 6 patients (3.37%) had a diagnosis of myelitis. Sixty two patients of 109 (55.9%) were classified as having meningitis due to undetermined etiologies. Enterovirus was the most frequent cause of viral meningitis in 21 patients (19%), followed by VZV in 16 patients (14.4%). HSV-1/-2 was identified in 7 patients (6.3%). One case was related to infection by parainfluenza virus based on the detection of viral DNA. In addition, one case of aseptic meningitis was due to Cryptococcus, based on detection by agglutinin test; bacteria were isolated in blood and CSF culture in one patient which was related to disseminated strongylodiasis.

There was a male predominance in VZV meningitis. Skin rash was noted in only 6/16 (37.5%) patients with VZV meningitis.

#### 3.1.2. Encephalitis

Thirty patients out of sixty-three (47.6%) were classified as encephalitis due to undetermined etiologies. The most frequent cause of encephalitis was HSV in 11 patients (17.5%), followed by WNV in 10 patients (15.9%). VZV was identified in 8 patients (12.7%) and 3/8 with herpetic rash (37.5%). In two cases of encephalitis enterovirus was identified, one case was due to influenza A, and one case was due to mycoplasma pneumonia.

#### 3.1.3. Myelitis

Myelitis was diagnosed in six patients; VZV was identified in 4/6 patients and 2/4 with herpetic rash. The other two patients were classified as myelitis due to undetermined etiology.

Clinical characteristics of patients with CNS VZV syndromes, treatment, and outcome are described in Table 1.

### 3.2. Treatment and Outcome (Table 2)

Most patients 13/16 (81.2%) patients with VZV meningitis were treated by acyclovir for a mean of 11.5 days (range, 5–28; median 7). Mean time of hospital stay in patients treated with acyclovir was 9.6 days (range, 3–30; median 7). Three patients were not treated by acyclovir and the mean hospital stay was 3.7 days (range, 3-4; median 4). All patients with VZV meningitis were discharged with improvement and resolution of symptoms and none was readmitted with any neurological complaint.

Seven of eight patients with VZV encephalitis were treated with intravenous acyclovir, three improved, and 4 succumbed. Of the surviving patients, one was without residual neurologic sequelae. Average length of hospital stay was 17.7 days (range, 5–32; median 19.5).

All four patients with VZV myelitis were treated by acyclovir, three patients improved, and one was discharged without improvement. Median time of median length of hospital stay was 11.75 days (range, 6–16; median 12.5).

## 4. Discussion

This study highlights the relative importance of VZV in CNS infectious syndromes; it is amongst the most common viral causes, regardless of any association with a typical herpetic rash. In our series, VZV was second to enterovirus as the most common cause of meningitis, the third most common cause of encephalitis behind HSV and West Nile fever, and the most common cause of myelitis. Notably, a significant percentage of each of these three syndromes was without a diagnosed cause. It is possible that wider usage of the BiofireFilmarray meningitis encephalitis (ME) panel, will enable the diagnosis of rare human herpesvirus 6 (HHV-6) and human parechovirus (HPeV) in adults, and thus the rate of undiagnosed causes will be reduced [16].

VZV was the most common viral etiology in a collaborative study from Finland 1995-1996; however, this study included children and patients with chickenpox and shingles as well. The viral etiologies were defined by molecular tests of CSF but also by serologic criteria of CSF and serum. The most common neurologic syndrome in this series was encephalitis [15].

The relative role of VZV in encephalitis varies widely 1.5%–18% [1, 6, 8, 10, 11] as opposed to its relative role in meningitis 11%–14% [3, 7, 8]. The variability can be ascribed to the difference in the methodology used for VZV testing and the difference in the target population. The cause of encephalitis in our series was undetermined in 47.6% and of meningitis in 55.9%, both figures are in accord with other studies [1–4, 7, 10].

The relative role of VZV amongst other viral causes in myelitis was not described in a series of 31 patients, rash was described in 45% of these patients, and 17/31 (54.8%) were immunocompromised [17].

Immunodeficiency was not a predominant underlying factor in our series. Medical background of relevance for possible immunocompromised state was available for all 28 patients with VZV CNS infection in our series. Three patients with meningitis were immunosuppressed (one with HIV, one with lung cancer under chemotherapy, and one with Waldenstrom macroglobulinemia), and skin rash was noted in two of them. VZV reactivation is commonly associated with immunosuppression. VZV with shingles is often associated with CSF abnormalities up to 61% in one series [18]. The association of VZV with immunosuppression is not apparent in our series, in part at least, due to the fact, that bone marrow or solid organ transplantation is not performed in this secondary medical center.

VZV CNS infection was age associated; in our series, VZV meningitis was more common in younger individuals; the average age was 41, while for encephalitis average age was 72. The association with age was in accord with previous series [1, 3, 6, 7, 11–14, 19].

CNS disease caused by VZV was more common in men than in women, 68.75% for meningitis and 75% for encephalitis. The association of VZV CNS infection with gender, shows a slight preponderance of men in VZV CNS infections [1, 2, 6, 7, 12–14, 19].

A typical herpetic rash in VZV-related CNS disease was observed in only 11/28 (39%) of cases in this series. This finding of a significant rate of VZV CNS infection without a rash is in accord with most series that assessed the presence of rash in VZV-related CNS infectious syndromes [2, 4, 5, 7–10, 12–14]. A relatively large study from Finland investigated 174 patients (including children) with acute CNS complications of VZV including chickenpox and shingles. The denominator for percent of herpetic rash in CNS infections then, was all cases with proven VZV infections with CNS involvement rather than a predominant CNS syndrome excluding chickenpox and shingles as in our series. In that series from Finland herpetic rash was absent in 27% (25/92) of encephalitis, 65% (17/26) of meningitis, and absent in all 4 cases with myelitis [20].

In our series, most VZV meningitis 13/16 (81.25%) cases were treated with acyclovir; nevertheless, all patients were discharged with complete resolution or significant improvement in symptoms, and none was readmitted to our hospital. A longer length of stay (LOS) was expected from a course of treatment and was similar to other series [3, 7, 12].

Our series cannot provide evidence in favor of acyclovir therapy and the data from other series is inconclusive as well [3, 12, 21]. The role of acyclovir therapy for VZV meningitis is unknown and has not yet been established through randomized controlled trials.

Evidence for the effectiveness of acyclovir treatment of VZV encephalitis is also inconclusive. This is due to the small number of patients, their advanced age and multiple comorbidities and due to a high rate of mortality (either directly or due to secondary complications) whether treatment is started early or not. In our series, the fatal outcome in 4/7 (57%) was similar to other series. Guidelines advocate early treatment with acyclovir for VZV encephalitis [2, 4, 6, 12, 14].

The incidence of myelitis in our study was low and therefore it was not possible to draw any conclusions as was the case for VZV-related myelitis in one series [17].

A recent, relatively large series of 128 patients from a large tertiary medical center in Israel described the significance and characteristics of 125 patients with VZV detection by PCR in CSF samples. This series stems from tracing all VZV cases detected in CSF samples, without comparison to other common viral causes. Therefore it does not determine the relative role of VZV amongst other causes of CNS infectious syndromes. In that series the case for CSF testing for VZV is not determined. VZV testing in CSF was requested following a presentation of chickenpox or shingles that was accompanied or followed by any neurologic syndrome, rather than a neurologic syndrome that mandates CSF tests, as in our series, regardless of rash. Thus, an increased rate of rash 79%, higher than in our series is not surprising. Yet, it was concluded that absence of rash does not exclude VZV as the cause of neurological symptoms [22].

The present study has several limitations. First, this study was retrospective in design, thus relying on patients' clinical records. Difficulties collecting complete clinical data may have resulted in subsequent bias. Second, the number of patients is not high enough for statistical analysis and reasoning. Third, it is a single-center study and caution is needed when interpreting the results in different environments. Nevertheless, the approach we took of molecular tests for viral causes of aseptic CSF from patients with neurologic presentation, may yield a reliable source for the relative role of VZV with and without rash in patients that present with CNS infectious syndromes. Wider application of this approach may yield better characterization of VZVs role in CNS infectious syndromes.

In conclusion, our results showed that VZV is a significant etiology of viral CNS infectious syndrome. Despite the development of the PCR technique, the etiology is unknown in ∼ 50% of patients. A typical rash appears at any point in only 39% of cases. The effect of antiviral treatment in patients with VZV meningitis and encephalitis is still inconclusive.

## Figures and Tables

**Table 1:** Clinical characteristics of patients with CNS VZV syndromes for each patient.

Patient no.	Main CNS syndrome	Gender	Age	LOS	Clinical signs				CSF			History		Brain lesion (CT/CTA/MRI)	PCR VZV lesion	PCR VZV CSF	Antiviral treatment		Death
					Skin rash	Fever	Headache	Meningeal sings	WBC	Mononuclear (%)	Protein (mg/dL)	CNS∗	Immunosuppression				Acyclovir	DOT	
1	Meningitis	F	39	5	+	+	+	−	640	95	225	−	−	−	−	+	+	7	−
2	Meningitis	F	34	3	+	−	+	+	296	100	137	−	−	−	+	+	+	15	−
3	Meningitis	M	42	5	+	−	+	−	250	100	124	−	−	−	−	+	+	9	−
4	Meningitis	F	17	5	+	+	+	+	80	98	72	−	−	−	−	+	+	6	−
5	Meningitis	M	81	9	+	−	−	+	20	98	39	−	+	−	−	+	+	6	−
6	Meningitis	M	67	17	+	−	−	−	34	92	31	+	+	−	−	+	+	23	−
7	Meningitis	M	19	3	−	+	+	+	150	98	126	−	−	−	−	+	−	0	−
8	Meningitis	M	46	4	−	−	+	−	200	100	120	−	−	−	−	+	−	0	−
9	Meningitis	M	19	7	−	+	+	+	7	NA	36	−	−	−	−	+	+	7	−
10	Meningitis	M	33	6	−	+	+	+	50	98	85	−	−	−	−	+	+	7	−
11	Meningitis	F	32	4	−	+	+	−	695	99	120	−	−	−	−	+	−	0	−
12	Meningitis	F	64	7	−	+	+	−	261	97	116	−	−	−	−	+	+	7	−
13	Meningitis	M	74	7	−	+	−	−	320	86	304	−	−	−	−	+	+	14	−
14	Meningitis	M	24	14	−	+	+	−	489	100	219	−	−	−	−	+	+	16	−
15	Meningitis	M	22	10	−	+	+	−	186	98	165	−	−	−	−	+	+	5	−
16	Meningitis	M	49	30	−	−	+	−	45	100	118	−	+	−	−	+	+	28	−
17	Encephalitis	M	59	7	+	+	+	−	0	NA	67	−	−	−	−	+	+	13	−
18	Encephalitis	M	72	8	+	+	−	−	42	80	83	+	−	+	−	+	+	7	+
19	Encephalitis	M	89	5	+	−	−	−	NA	NA	NA	+	−	−	+	NA	+	4	+
20	Encephalitis	F	87	26	−	+	−	−	0	NA	53	+	−	+	−	+	+	6	+
21	Encephalitis	M	36	17	−	+	−	−	4	NA	41	+	−	−	−	+	+	17	−
22	Encephalitis	M	92	22	−	+	−	−	25	NA	152	−	−	−	−	+	−	0	−
23	Encephalitis	F	66	32	−	+	−	−	270	95	481	+	−	+	−	+	+	32	−
24	Encephalitis	M	73	29	−	+	−	−	1	NA	32	−	−	−	−	+	+	5	+
25	Myelitis	F	63	9	+	−	−	−	0	NA	28	−	−	+	+	−	+	8	−
26	Myelitis	F	61	16	+	−	−	−	3	NA	30	−	−	+	−	+	+	17	−
27	Myelitis	M	51	6	−	−	−	−	40	100	54	−	−	+	−	+	+	7	−
28	Myelitis	F	53	16	−	−	−	−	35	100	339	−	−	+	−	+	+	9	−

Abbreviations: CNS, central nervous system; CSF, cerebrospinal fluid; CT, computed tomography; CTA, computed tomographic angiography; DOT, days of treatment; LOS, length of stay; NA, not available; No, number; PCR, polymerase chain reaction; VZV, varicella zoster virus; WBC, white blood cells.

^∗^–CNS disorders—demention, status post CVA, Parkinson, epilepsy.

**Table 2:** Clinical characteristics of patients with CNS infectious syndromes and VZV in CSF for each syndrome.

	Meningitis *N* = 16	Encephalitis *N* = 8	Myelitis *N* = 4	Overall *N* = 28
Age (years)	41.38	71.75	57.83	
Male, *n* (%)	11 (68.75)	6 (75)	1 (25)	18 (64.29)
Female, *n* (%)	5 (31.5)	2 (25)	3 (75)	10 (35.71)
Immunosuppression, *n* (%)	3 (18.75)	0 (0)	0 (0)	3 (10.71)
Length of stay (days, average)	8.5	18.25	11.75	
Rash, *n* (%)	6 (37.5)	3 (37.5)	2 (50)	11 (39.28)
Fever *n* (%)	10 (62.5)	7 (87.5)	0	17 (60.71)
Headache *n* (%)	13 (81.25)	1 (12.5)	0	14 (50)
Meningeal signs *n* (%)	6 (37.5)	0	0	
Encephalitis symptoms *n* (%)	0	8	4∗	
C-reactive protein (mg/dL, average) Normal range 0.0–0.5	0.48	7.2	0.5	
Cerebrospinal fluid analysis				
Mean white blood cells (cells/*μ*l)	232.7 Range 7–695	48.8 Range 0–270	19.5 Range 0–40	
Mean protein (mg/dL)	127.3	129.9	112.75	
Mean glycose (mg/dL)	60.2	99.9	70.25	

^∗^–Focal symptoms (paresis/paralysis).

## Data Availability

Data are available on request.

## Nomenclature

CNS: Central nervous system
CSF: Cerebrospinal fluid
VZV: Varicella zoster virus
HSV: Herpes simplex virus
WNV: West Nile virus
PCR: Polymerase chain reaction

## References

[1] Mailles A., Stahl J. P. (2009). Infectious Encephalitis in France in 2007: A National Prospective Study. *Clinical Infectious Diseases*.

[2] Glaser C. A., Honarmand S., Anderson L. J. (2006). Beyond Viruses: Clinical Profiles and Etiologies Associated With Encephalitis. *Clinical Infectious Diseases*.

[3] Jarrin I., Sellier P., Lopes A. (2016). Etiologies and Management of Aseptic Meningitis in Patients Admitted to an Internal Medicine Department. *Medicine (Baltimore)*.

[4] Tunkel A. R., Glaser C. A., Bloch K. C. (2008). The Management of Encephalitis: Clinical Practice Guidelines by the Infectious Diseases Society of America. *Clinical Infectious Diseases*.

[5] Venkatesan A., Geocadin R. G. (2014). Diagnosis and Management of Acute Encephalitis: A Practical Approach. *Neurology Clinical Practice*.

[6] Arruti M., Piñeiro L. D., Salicio Y., Cilla G., Goenaga M. A., López de Munain A. (2017). Incidence of Varicella Zoster Virus Infections of the Central Nervous System in the Elderly: A Large Tertiary Hospital-Based Series (2007–2014). *Journal of NeuroVirology*.

[7] Han S. H., Choi H. Y., Kim J. M., Park K. R., Youn Y. C., Shin H. W. (2016). Etiology of Aseptic Meningitis and Clinical Characteristics in Immune-Competent Adults. *Journal of Medical Virology*.

[8] Choi R., Kim G. M., Jo I. J. (2014). Incidence and Clinical Features of Herpes Simplex Viruses (1 and 2) and Varicella-Zoster Virus Infections in an Adult Korean Population With Aseptic Meningitis or Encephalitis. *Journal of Medical Virology*.

[9] Grahn A., Studahl M. (2015). Varicella-Zoster Virus Infections of the Central Nervous System-Prognosis, Diagnostics and Treatment. *Journal of Infection*.

[10] Granerod J., Ambrose H. E., Davies N. W. (2010). Causes of Encephalitis and Differences in Their Clinical Presentations in England: A Multicentre, Population-Based Prospective Study. *The Lancet Infectious Diseases*.

[11] Parisi S. G., Basso M., Del Vecchio C. (2016). Viral Infections of the Central Nervous System in Elderly Patients: A Retrospective Study. *International Journal of Infectious Diseases*.

[12] Becerra J. C., Sieber R., Martinetti G., Costa S. T., Meylan P., Bernasconi E. (2013). Infection of the Central Nervous System Caused by Varicella Zoster Virus Reactivation: A Retrospective Case Series Study. *International Journal of Infectious Diseases*.

[13] Kaewpoowat Q., Salazar L., Aguilera E., Wootton S. H., Hasbun R. (2016). Herpes Simplex and Varicella Zoster CNS Infections: Clinical Presentations, Treatments and Outcomes. *Infection*.

[14] Chamizo F. J., Gilarranz R., Hernández M., Ramos D., Pena M. J. (2016). Central Nervous System Infections Caused by Varicella-Zoster Virus. *Journal of NeuroVirology*.

[15] Koskiniemi M., Rantalaiho T., Piiparinen H. (2001). Infections of the Central Nervous System of Suspected Viral Origin: A Collaborative Study From Finland. *Journal of NeuroVirology*.

[16] Trujillo-Gómez J., Tsokani S., Arango-Ferreira C. (2022). Biofire Film Array Meningitis/Encephalitis Panel for the Aetiological Diagnosis of Central Nervous System Infections: A Systematic Review and Diagnostic Test Accuracy Meta-Analysis. *eClinicalMedicine*.

[17] Hung C. H., Chang K. H., Kuo H. C. (2012). Features of Varicella Zoster Virus Myelitis and Dependence on Immune Status. *Journal of the Neurological Sciences*.

[18] Haanpää M., Dastidar P., Weinberg A. (1998). CSF and MRI Findings in Patients With Acute Herpes Zoster. *Neurology*.

[19] Kim S. H., Choi S. M., Kim B. C. (2017). Risk Factors for Aseptic Meningitis in Herpes Zoster Patients. *Annals of Dermatology*.

[20] Koskiniemi M., Piiparinen H., Rantalaiho T. (2002). Acute Central Nervous System Complications in Varicella Zoster Virus Infections. *Journal of Clinical Virology*.

[21] Han J. Y., Hanson D. C., Way S. S. (2011). Herpes Zoster and Meningitis Due to Reactivation of Varicella Vaccine Virus in an Immunocompetent Child. *The Pediatric Infectious Disease Journal*.

[22] Kriger O., Dovrat S., Fratty I. S. (2024). Don’t Rash It! the Clinical Significance of Positive Varicella Zoster Virus PCR in Cerebrospinal Fluid of Patients With Neurological Symptoms. *Journal of Clinical Virology: The Official Publication of the Pan American Society for Clinical Virology*.

